# Glutaminolysis Was Induced by TGF-β1 through PP2Ac Regulated Raf-MEK-ERK Signaling in Endothelial Cells

**DOI:** 10.1371/journal.pone.0162658

**Published:** 2016-09-09

**Authors:** YanYan Guo, YuanJun Deng, XiaoQing Li, Yong Ning, XuePing Lin, ShuiMing Guo, MeiXue Chen, Min Han

**Affiliations:** Department of Nephrology, Division of Internal Medicine, Tongji Hospital, Tongji Medical College, Huazhong University of Sicence and Technology, Wuhan, Hubei, China; Beijing Key Laboratory of Diabetes Prevention and Research, CHINA

## Abstract

Vascular endothelial cells can survive under hypoxic and inflammatory conditions by alterations of the cellular energy metabolism. In addition to high rates of glycolysis, glutaminolysis is another important way of providing the required energy to support cellular sprouting in such situations. However, the exact mechanism in which endothelial cells upregulate glutaminolysis remains unclear. Here we demonstrated that protein phosphatase 2A (PP2A)-mediated Raf-MEK-ERK signaling was involved in glutaminolysis in endothelial cells. Using models of human umbilical vein endothelial cells (HUVECs) treated with transforming growth factor-β1 (TGF-β1), we observed a dramatic induction in cellular glutamate levels accompanied by Raf-MEK-ERK activation. By addition of U0126, the specific inhibitor of MEK1/2, the expression of kidney-type glutaminase (KGA, a critical glutaminase in glutaminolysis) was significantly decreased. Moreover, inhibition of PP2A by okadaic acid (OA), a specific inhibitor of PP2A phosphatase activity or by depletion of its catalytic subunit (PP2Ac), led to a significant inactivation of Raf-MEK-ERK signaling and reduced glutaminolysis in endothelial cells. Taken together, these results indicated that PP2A-dependent Raf-MEK-ERK activation was involved in glutaminolysis and inhibition of PP2A signals was sufficient to block Raf-MEK-ERK pathway and reduced glutamine metabolism in endothelial cells.

## Introduction

Mammalian cells fuel their growth and proliferation through the catabolism of two main substrates: glucose and glutamine. Many human cell lines, especially cancer cells, depend on a high rate of uptake and metabolism of glucose to maintain their viability [1]. In addition to altered glucose metabolism, glutaminolysis (catabolism of glutamine to ATP and lactate) has been demonstrated to be another important source of energy and plays critical roles in rapidly dividing cells and tumor cells [2, 3]. In glutaminolysis, glutamine is converted into glutamate and ammonia with the action of glutaminase. Glutamate is further catabolized into ATP, lipids, nucleotides or glutathione in the Krebs cycle, which provides materials and energy for cells.

Similar to tumor cells, evidence supporting that the high rate of energy metabolism in the vascular endothelium has also been described [4, 5]. It is noteworthy that endothelial cells in blood vessels are exposed to high levels of oxygen and other nutrients, and are more likely to encounter hypoxic and nutrient-deprived conditions in disease states. Thus, to adapt to excess oxygen as well as to a hypoxic environment, endothelial cells require a particular metabolic capacity for such fluctuations [5]. However, the exact mechanism in which endothelial cells deal with different biological situations by their metabolism remains unknown.

As the key and limit enzyme in catalyzing glutaminolysis, glutaminase is becoming an attractive target for tumor therapy and regarded as the new research direction [6]. Increased expression of glutaminase upregulated the glutaminolysis and produced more ATP and glutathione, resulting in protective roles in tumor cells from reactive oxygen species damage [7]. Inhibition of glutaminase reduced glutathione antioxidant capacity and increased apoptosis of tumor cells [8]. Humans express two glutaminase isoforms: kidney-type glutaminase (KGA) and liver-type glutaminase (LGA) from two closely related genes [9]. Although KGA is the first enzyme in catalyzing glutaminolysis and is important for promoting growth, the precise mechanism of its activation is not yet understood.

It was recently hypothesized that transforming growth factor-β (TGF-β) may activate glutaminase to enhance intracellular catabolism of glutamine [10]. Over the past several years, TGF-β has been demonstrated to play critical roles in cell growth, differentiation, apoptosis, migration and the matrix formation [11–15]. TGF-β signaling pathways, including classic Smads pathway, non-Smads pathways (including the MEK/ERK, PI3K and p38 MAPK pathways) and the NF-κB pathway, activate or act synergistically in the regulation of glutaminase. The study by Andratsch M revealed that TGF-β1 activated multiple signaling pathways in LLC-PK1-FBPase+ cells and enhanced the expression of glutaminase [16]. Another study showed that the phosphate-dependent activity of KGA is regulated by Raf-MEK-ERK signaling pathway and protein phosphatase 2A (PP2A) in cancer cells [17]. Notably, PP2A is a ubiquitous and conserved serine/threonine phosphatase and is composed of a catalytic subunit (PP2Ac), a structural subunit (PP2Aa) and a variable regulatory subunit (PP2Ab). Evidence demonstrated that PP2A could dephosphorylate all kinases of the ERK cascade including c-Raf, MEK, ERK [18–24].

In present study, TGF-β1 was tested for the ability to induce glutaminolysis in endothelial cells and the exact mechanisms were explored. Our results indicate that TGF-β1 promotes glutamine metabolism in endothelial cells, and the activity of KGA has important relation with the activation of PP2A-mediated Raf-MEK-ERK signaling pathway.

## Materials and Methods

### Cell cultures

Human umbilical vein endothelial cells (HUVECs, ATCC, USA) were cultured in RPMI-1640 (Gibco, USA) supplemented with 10% fetal bovine serum (FBS, HyClone, USA) at 37°C in 5% CO_2_. When 30% confluent, cells were cultured in serum-free medium before recombinant human TGF-β1 (10 ng/ml, Peprotech, NJ, USA) treatment. In some experiments, cells were pretreated with PP2A inhibitor OA (10 nM, Sigma, USA) or MEK 1/2 inhibitor (U0126, 10 μM, Sigma, USA) for 30 min before the treatment with TGF-β1. For all cell stimulations, n≥3 independent experiments were performed.

### Transfection of siRNA

2×10^5^ of HUVECs per well were seeded in 6-well plates. when they reached 60% confluence, the cells were transiently transfected with PP2Ac siRNA or control siRNA (Invitrogen, USA) by using lipofectamine 2000 transfection reagent (Invitrogen, USA) in Opti- MEMI-reduced serum medium (Invitrogen, USA) without FBS and antibiotics according to the manufacturer′s instructions. After six-hour transfection, the cells were treated with TGF-β1 for 15 min or 60 min and then cell viability was detected.

### Western blotting

After treatment, cells were harvested and lysed with RIPA buffer supplemented with protease inhibitor cocktail. Protein concentration was measured by using BCA assay kit (Pierce, USA). 60 micrograms of protein was subjected to 10% SDS-PAGE and then transferred to polyvinylidene difluoride membranes (Roche, Switzerland). The blocked membranes were incubated with primary antibodies against phospho-c-Raf (Ser-259) (CST, USA), c-Raf (CST, USA), phospho- ERK1/2 (Thr202/Tyr204) (CST, USA), ERK1/2 (CST, USA), PP2A catalytic subunit (CST, USA), KGA (Proteintech, USA), β-actin (Wuhan Goodbio, China), glyceraldehyde-3-phosphate dehydrogenase (GAPDH, Wuhan Goodbio, China) at 4°C overnight, then incubated with horseradish peroxidase-conjugated secondary antibodies anti-rabbit HRP and anti-mouse HRP antibodies (Wuhan Goodbio, China) for 1 h at 37°C. The immunolabeled proteins were detected by enhanced chemiluminescence (Pierce, USA). The density of the bands was analyzed by Quantity One software (Bio-Rad).

### PP2A activity assay

Protein Ser/Thr phosphatase activity was assayed photometrically using the Serine/Threonine Phosphatase Assay System (Promega, USA) according to the manufacturer’s instructions. TGF-β1 treated cells were lysed by RIPA buffer supplemented with protease inhibitor cocktail. 250 μl of the supernatant was passed through the prepared Sephadex G-25 spin column to remove free phosphate. The sample was incubated with reaction solution at 37°C for 15 min. Then the reaction was terminated with Molybdate Dye/Additive mixture at room temperature for 15 min. The plate was read at 600 nm to calculate the activity of PP2A.

### Intracellular glutamate test

The concentration of intracellular glutamate was detected using the Amplex^®^Red Glutamic Acid / Glutamate Oxidase Assay Kit (Invitrogen, USA) according to the manufacture′s procedure. TGF-β1-treated cells were lysed by RIPA buffer supplemented with protease inhibitor cocktail. 50 μl of supernatant or H_2_O_2_ positive control was diluted by 1 × reaction buffer, then 50 μl of Amplex^®^Red reagent / HRP / glutamate oxidase / glutamate—pyruvate transaminase / alanine working solution was added to start the reaction. The sample was incubated at 37°C for 30 min in darkness. The plate was measured at 530 / 590 nm to calculate the concentration of intracellular glutamate.

### Cell migration assay

Cells plated on 6 well-plates grew up to 95% confluence and then were washed with PBS or serum free media to remove traces of stimulation factors and suspending death cells. The scratch was made using a 10 μL tip and images were captured from 0 hour to 48 hours using an inverted microscope. The percentage of gap distance was measured with Image Pro Plus software using the formula ((area of scratch a 0 hr—scratch at 48 hr) / scratch at 0 hr))*100.

### Statistical Analyses

Data were analyzed using standard statistical methods, including linear regression, the t test and one-way ANOVA using SPSS (version 15.0). Data were expressed as the mean ±SEM. P<0.05 was considered statistically significant.

## Results

### 1. Glutaminolysis is induced by TGF-β1 in HUVECs

**Fig 1:**
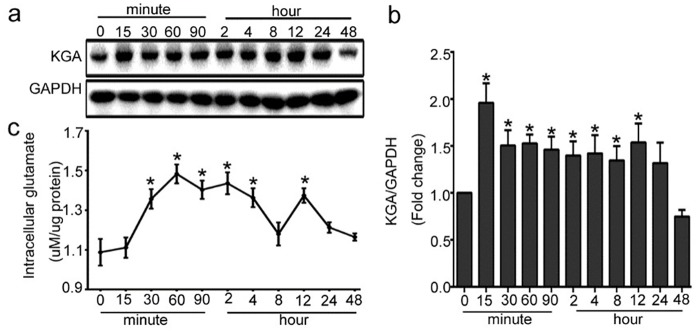
Glutaminolysis is induced by TGF-β1 in HUVECs. The cells were treated with 10ng/ml of TGF-β1 for different indicated periods, from 15 minutes to 48 hours. (a) KGA protein expression was up-regulated by TGF-β1. Representative western blot of KGA in each group is shown. (b) Densitometry analysis of KGA protein expression was plotted (n≥3). (c) Intracellular glutamate level in the medium was analyzed using theAmplex^®^Red Glutamic Acid / Glutamate Oxidase Assay Kit (n≥3). *P<0.05 compared to control group. Bars represented means ± SEM.

To explore the change of glutaminolysis in HUVECs treated with TGF-β1, we detected the protein expression of KGA, the key enzymes of glutamine metabolism, and the concentration of intracellular glutamate. The expression of KGA was significantly up-regulated in HUVECs from 15 min after the cells were treated with 10 ng/ml of TGF-β1, and lasted for almost 12 h, then the KGA protein expression was gradually decreased. (Fig 1a and 1b). Consistent with the change of KGA, the intracellular glutamate was gradually increased from 15min with TGF-β1 stimulation, peaked at 60 min, then kept in a high level until 12h (Fig 1c). Together, these data suggested that TGF-β1 induced glutaminolysis in HUVECs.

### 2. Raf-MEK-ERK signaling is involved in KGA activation in HUVECs

**Fig 2:**
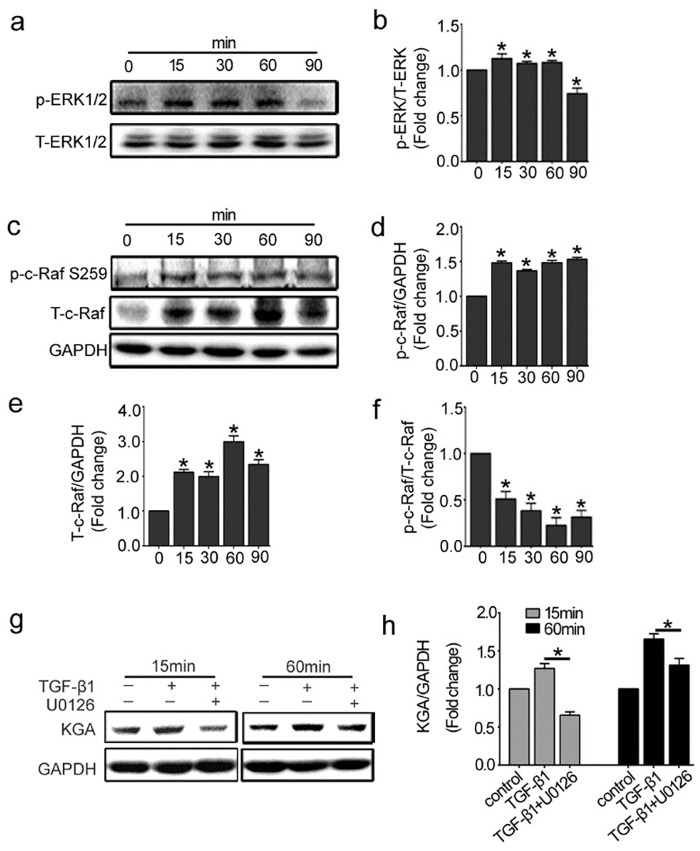
Raf-MEK-ERK signaling pathway is involved in KGA activation in HUVECs. The cells were treated with 10ng/ml of TGF-β1 for different indicated periods, from 15 minutes to 90 minutes. (a, c) Representative western blot of p-ERK1/2, T-ERK1/2, p-c-Raf (Ser 259) and T-c-Raf in each group was shown. (b, d, e, f) The histogram are normalized to a GAPDH control and showed the ratio of p-ERK1/2 to T-ERK1/2 and p-c-Raf to T-c-Raf (n≥3). (g, h) HUVECs were pretreated with MEK 1/2 inhibitor (U0126, 10μM) for 30 min. KGA protein expressions were detected by western blot with densitometry analysis (n≥3). *P<0.05 compared to control group. Bars represented means ±SEM.

Previous work has demonstrated that the Raf-MEK-ERK pathway can regulate KGA activity in cancer cells [17]. We therefore investigated whether the Raf-MEK-ERK pathway was associated with KGA and glutamine metabolism in endothelial cells. Firstly, we detected the expression of phosphorylated-ERK1/2 (p-ERK1/2) and Total-ERK1/2(T- ERK1/2) in response to TGF-β1 in HUVECs at different time points. Fig 2a showed that the expression of p-ERK1/2 increased from 15 min and sustained for 60 min. It meant the MEK/ERK pathway activation. Then we detected the expression of c-Raf, the upstream signal molecule of ERK1/2. In resting cells, Serine 259 on c-Raf usually keeps hyperphosphorylatd as an inhibitory phosphorylation site. Once this site is dephosphorylated, c-Raf is activated [25–27]. As shown in Fig 2c, phosphorylated-c-Raf (Ser 259) and total-c-Raf were up-regulated from 15 min to 90 min after TGF-β1 incubation. Notably, the rate of phosphorylated-c-Raf (Ser 259) to total-c-Raf decreased and reached the bottom at 60 min, which meant more c-Raf was activated. These results indicated that Raf-MEK-ERK signaling was activated under the stimulation of TGF-β1 in HUVECs. To further investigate whether c-Raf-MEK1/2-ERK1/2 signaling pathway is involved in KGA activation, we pretreated HUVECs with U0126 (10 nmol/L), the specific inhibitor of MEK1/2, prior to TGF-β1 stimulation. The data showed that the KGA protein expression was inhibited by U0126 at 15 min and 60 min after TGF-β1 incubation (Fig 2g). Taken together, these results indicated that the activation of c-Raf-MEK-ERK signaling pathway participated in the regulation of KGA in HUVECs.

### 3. PP2A activity is induced by TGF-β1, whereas PP2Ac protein expression is not changed

**Fig 3:**
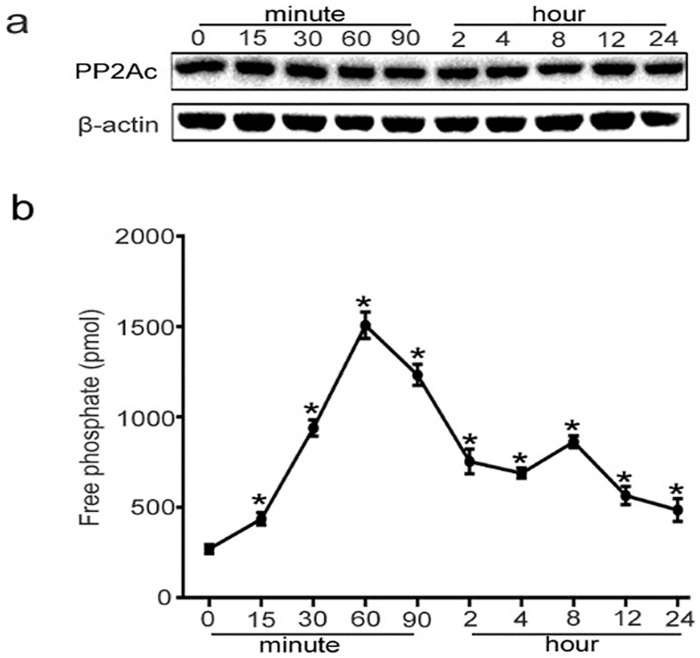
PP2Ac protein expression and PP2A activity in TGF-β1-treated HUVECs. The cells were treated with 10ng/ml of TGF-β1 for different indicated periods, from 15 minutes to 24 hours. (a) PP2Ac protein expression was measured by Western blot analysis. (b) PP2Ac activity was detected by using the Serine/Threonine Phosphatase Assay System in TGF-β1-treated HUVECs (n≥3). *P<0.05 compared to control group. Bars represented means ± SEM.

PP2A exhibits diverse regulatory roles in the control of various MAPK signal transduction pathways [21–24]. And it was proven that PP2A induced activation of Raf kinase via dephosphoryation of serine 259[18–20]. Therefore, we investigated whether PP2A contributes to glutamine metabolism in endothelial cells via Raf-MEK-ERK signaling. The results showed that PP2Ac (the catalytic subunit of PP2A) protein level was not changed during the treatment of TGF-β1 (Fig 3a). However, its activity increased from 15 min and reached the peak at 60 min, and then gradually reduced to the basal level after 24 h (Fig 3b).

### 4. OA prevents the activation of Raf-MEK-ERK pathway induced by TGF-β1

**Fig 4:**
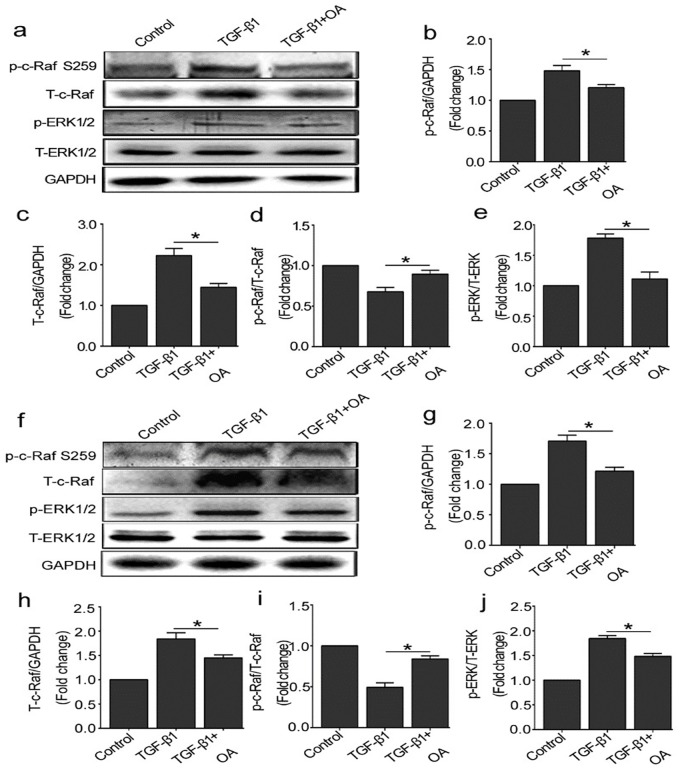
OA prevents the activation of the Raf-MEK-ERK pathway induced by TGF-β1. (a) The cells were treated with OA (10 nM) for 30 min before TGF-β1 (10 ng/ml) treatment. Representative western blot of p-ERK1/2, T-ERK1/2, p-c-Raf (Ser 259) and T-c-Raf in each group were shown. (b, c, d, e) The histograms were normalized to a GAPDH control and showed the ratio of p-ERK1/2 to T-ERK1/2 and p-c-Raf to T-c-Raf. (n≥3). (f) The cells were pretreated with OA (10 nM) for 30 min and then treated with TGF-β1 (10 ng/ml) for 60 min. Representative western blot of p-ERK1/2, T-ERK1/2, p-c-Raf (Ser 259) and T-c-Raf in each group were shown. (g, h, i, j) The histograms were normalized to a GAPDH control and showed the ratio of p-ERK1/2 to T-ERK1/2 and p-c-Raf to T-c-Raf. (n≥3). *P<0.05 compared to control group. Bars represented means ±SEM.

To further investigate the role of PP2A on Raf-MEK-ERK signaling pathway in TGF-β1 treated HUVECs, the cells were pretreated with okadaic acid (OA 10 nM), a specific inhibitor of PP2A phosphatase activity, prior to TGF-β1 treatment. Raf-MEK-ERK signaling molecules were detected at 15 min or 60 min after TGF-β1 stimulation. As shown in Fig 4, compared with TGF-β1 group, OA inhibited p-c-Raf (Ser 259) and total-c-Raf protein expression induced by TGF-β1, and the rate of phosphorylated-c-Raf to total-c-Raf increased. Consistently, the expression of p-ERK was also prevented after OA pretreatment. These results indicated that PP2A activated Raf-MEK-ERK signaling in response to TGF-β1 and blockade of PP2A could inhibit this process.

### 5. PP2Ac depletion by specific siRNA inhibits the activation of Raf-MEK-ERK signaling in response to TGF-β1

**Fig 5:**
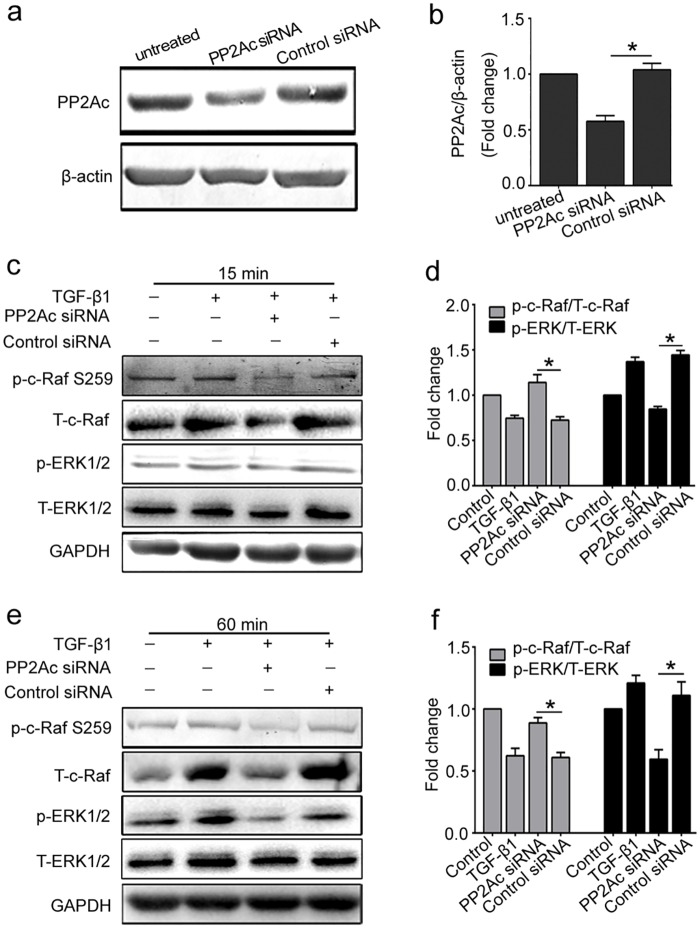
Knockdown of PP2Ac by siRNA prevents the activation of Raf/MEK/ERK pathway in response to TGF-β1. (a) HUVECs were transiently transfected with 100 nmol/L of PP2Ac siRNA or control siRNA for 12 h. PP2Ac protein expression was detected to confirm the knockdown effect. A representative western blot was shown. (b) Densitometry analysis of PP2Ac protein expression was plotted (n≥3). (c, d, e, f) After transfection of PP2Ac siRNA, cells were incubated with TGF-β1 for 15 min and 60 min, respectively. A representative western blot of p-c-Raf (Ser 259), T-c-Raf, p-ERK 1/2, T-ERK 1/2 in each group were shown. The histograms showed the ratio of p-c-Raf/T-c-Raf and p-ERK/T-ERK (n≥3).*P<0.05 compared to control group. Bars represented means ±SEM.

To further illustrate the role of PP2Ac on activation of Raf-MEK-ERK signaling in TGF-β1 stimulated HUVECs, we applied small interfering RNAs (siRNA) to directly and specifically knock down PP2Ac and confirmed the effect by Western blot analysis(Fig 5a). Compared with TGF-β1, depletion of PP2Ac inhibited the expression of c-Raf, and the hyper-phosphorylation state of Ser 259 on c-Raf was maintained. The downstream molecule of this signaling pathway, ERK1/2, was also dephosphorylated after PP2Ac depletion. In general, all these results further confirmed that PP2A positively regulates the activation of Raf-MEK-ERK signaling in TGF-β1-induced glutaminosis in HUVECs.

### 6. TGF-β1 induced-glutaminolysis accelerates endothelial migration and PP2A-Raf-MEK-ERK pathway is involved in this process

**Fig 6:**
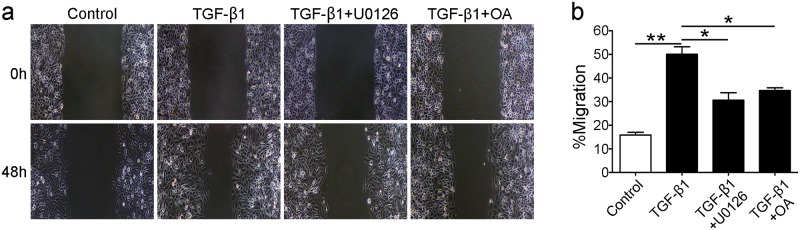
TGF-β1-induced glutaminolysis accelerates endothelial migration and PP2A-Raf-MEK-ERK pathway is involved in this process. The cells were pretreated with U0126 (10μM) and OA (10 nM) for 30 minutes before TGF-β1 (10 ng/ml) stimulation and then cultured for 48 hours. (a) Cell migration was promoted by TGF-β1, and inhibition of PP2A-Raf-MEK-ERK pathway inhibited cells sprouting. (b) Bar graphs present the gap distance calculated by Image Pro Plus Software.**P<0.01 versus the control group, and *P<0.05 versus the TGF-β1 group. Bars represented means ±SEM.

To investigate the role of TGF-β1 and PP2A-Raf-MEK-ERK signaling pathway in HUVECs migration, cells were pretreated with OA (10nM) and U0126 (10μM) prior to TGF-β1(10ng/mL) treatment. As shown in Fig 6, compared with control group, TGF-β1 promoted cells migration. OA or U0126 pretreatment could inhibit cell migration compared with TGF-β1 group. These results indicated that TGF-β1 induced glutaminolysis promotes endothelial cell sprouting and PP2A-Raf-MEK-ERK pathway were involved in this process.

## Discussion

**Fig 7:**
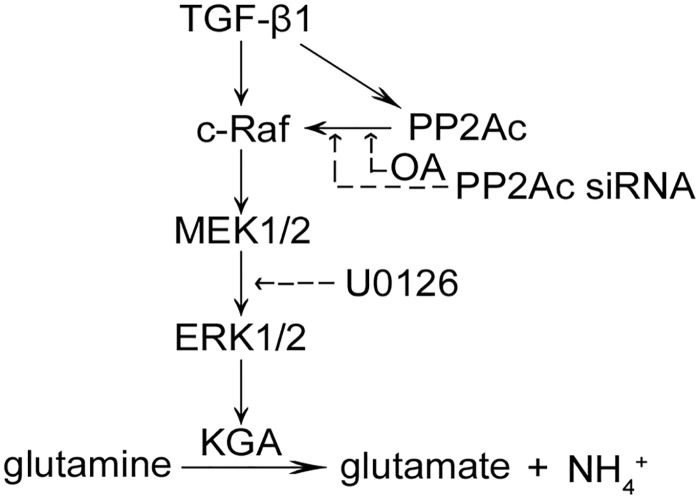
Diagram of the mechanism by which glutaminolysis is induced by TGF-β1 through PP2Ac regulated Raf-MEK-ERK signaling in endothelial cells. The solid arrows mean stimulation, and dotted arrows mean inhibitory effect.

In this study, we identified a critical role for PP2A in TGF-β1-induced glutaminolysis in endothelial cells (ECs) via Raf-MEK-ERK pathway. Inhibiting PP2A activation by its specific inhibitor or by depletion of its catalytic subunit (PP2Ac) prevented the activation of Raf-MEK-ERK signaling and reduced KGA expression induced by TGF-β1 (Fig 7).

Accumulated evidence demonstrated that glutaminolysis is another hallmark of cancer cells in addition to aberrant glucose metabolism [28] and is closely related to cancer and nerve system diseases [29–33]. Our data indicated that there also existed glutamine metabolic changes in the pathogenesis of endothelial cells mediated by TGF-β1. We also investigated the changes of ATP level in TGF-β1 induced glutaminolysis in HUVECs. Our results suggested that TGF-β1 induced glutaminolysis, which changed ATP level in endothelial cells (S1 Fig). Glutaminase is the first and rate-limiting enzyme in glutaminolysis and plays an essential part in glutaminolysis through catalyzing the conversion of glutamine to glutamate. Two different but closely related genes, GLS1 (glucan synthesis gene 1) and GLS2 (glucan synthesis gene 2) are responsible for synthesizing glutaminase. GLS2 is liver-type glutaminase (LGA) detected mainly in liver, while GLS1 is kidney-type glutaminase including three isoforms through alternative splicing: kidney-type glutaminase isoform (KGA), glutaminase C isoform (GAC) and glutaminase M isoform (GAM). KGA is expressed in many organs such as kidney, brain and intestine [34] and is often upregulated in cancer and is thus an attractive anti-cancer drug target [35]. The research about KGA in cancer cells is widespread and sufficient, but in our study, we tried to explore whether KGA participated in the pathogenesis of endothelial cells. In present study, the expression of KGA and intracellular glutamate generation were upregulated after TGF-β1 stimulation. Our data showed exactly that exposure to TGF-β1 increased KGA protein expression in HUVECs, which peaked at 15min and maintained at high levels for 12h (Fig 1a). To strengthen the results, we analyzed glutamate production, a metabolic product of glutamine catalyzed by KGA. We observed increased levels of glutamate in response to TGF-β1 treatment, which was consistent with the change of KGA (Fig 1c). Together, these factors indicated that KGA activated in endothelial cells after treatment with TGF-β1 and catalyzed the conversion of glutamine to glutamate, which resulted in glutaminolysis in ECs. There is evidence that the kinetic properties of KGA is affected by glutamate and the affinity of KGA and glutamine can be strongly inhibited by levels of glutamate [36]. In this study, we observed obvious decline of glutamate at 8 h and the expression of KGA and glutamate level were both up-regulated at 12 h, which meant that a balance was reached between KGA and glutamate.

High levels of KGA and glutamate were promoted by a series of signal transduction in cells and preventing the signals would significantly hinder cancer cell growth and proliferation. Evidence demonstrated the Raf-MEK-ERK pathway was aberrantly activated in human diseases and played a critical role in the regulation of KGA in cancer cell metabolism. In this study, The Raf-MEK-ERK pathway, a three-tier kinase cascade, could be spatially coordinated in regulating glutaminolysis in endothelial cells. Our data showed that activated c-Raf regulates the phosphorylation of downstream signaling molecules MEK1/2 and ERK1/2 and induced the activation of the pathway to relay signals. However, the activation mechanism of c-Raf is complex and is incompletely understood. It was reported that phosphorylated serine 259 of c-Raf provides a binding site, which inhibits the recruitment of c-Raf to the membrane and the interaction with Ras. So phosphorylation of serine 259 could inhibit the c-Raf kinase and dephosphorylation of c-Raf released its activity. Here, we showed that TGF-β1 increased the expression of phosphorylated-c-Raf Ser259 and total-c-Raf protein in endothelial cells. Of note, the rise in total-c-Raf protein is more rapid than that in p-c-Raf, which resulted in a reduction of the ratio of phosphorylated-c-Raf to total-c-Raf. Therefore, c-Raf activated and finally induced the activation of ERK1/2. As shown in Fig 2, both ERK1/2 and c-Raf kinases activated within 90 min after TGF-β1 treatment and their changing trend kept consistent. Whether other TGF-β1 signaling pathways are involved in regulating the expression of KGA needs more research.

C-Raf kinases activation depends on its dephosphorylation status, however, upstream regulating molecules that cause c-Raf dephosphorylation remain poorly understood. Herein we focused on the role of PP2A in the activation of c-Raf in present study. PP2A accounts for as much as 1% of total cellular proteins and represents the major portion of serine/threonine phosphatase activity in most tissue and cells [37]. We further explored to determine whether TGF-β1-induced Raf-MEK-ERK activation depended on PP2Ac positive regulation. Inhibition of PP2A activity by its specific inhibitor or transfection with PP2Ac siRNA prevented the activation of Raf-MEK-ERK pathway induced by TGF-β1 (Figs 4 and 5). Thus, we hypothesize that PP2A promotes dephosphorylation of serine 259 site in c-Raf and actives c-Raf-MEK-ERK signaling. Our results were consistent with a recent study, which demonstrated that PP2A functions as a positive regulator of Raf-MEK1/2-ERK1/2 signaling [38].

In summary, our results indicated that there exists glutamine metabolic changes in the physiological and pathological process of endothelial cells mediated by TGF-β1. Meanwhile, our study provided detailed insights into the regulation of KGA activity by Raf-MEK-ERK signaling module, and PP2A dephosphorylation-dependent regime in glutaminolysis induced by TGF-β1. Inhibiting PP2A-PP2Ac signals was sufficient to significantly block Raf-MEK-ERK activation and reduce glutamine metabolism in endothelial cells, which provided a new direction for the research in endothelial dysfunction.

## Supporting Information

S1 Fig ATP level in TGF-β1 induced glutaminolysis in HUVECs. Intracellular ATP level was measured in response to TGF-β1 (10 ng/ml) for 15min to 24 hours. n≥3/group, *P<0.05, * * P<0.01 compared with control group. Bars represented means ±SEM.
(PDF)
