# Supplementary material for: Glutaminolysis Was Induced by TGF-β1 through PP2Ac Regulated Raf-MEK-ERK Signaling in Endothelial Cells
Source: PLoS One. 2016 Sep 9;11(9):e0162658. doi: 10.1371/journal.pone.0162658 (PMC5017743; doi:10.1371/journal.pone.0162658)
Supplement: S1 Fig — Intracellular ATP level was measured in response to TGF-β1 (10 ng/ml) for 15min to 24 hours. n≥3/group, *P<0.05, * * P<0.01 compared with control group. Bars represented means ±SEM. (PDF) [file pone.0162658.s001.pdf]

Supporting Information

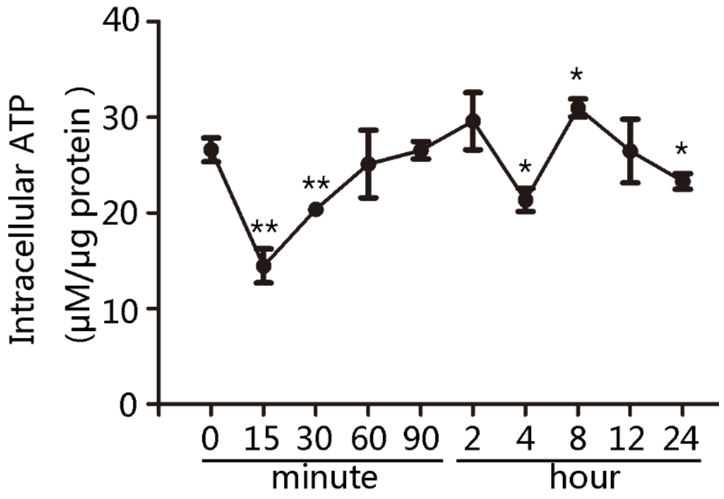

S1 Fig. ATP level in TGF-β1 induced glutaminolysis in HUVECs.

Intracellular ATP level was measured in response to TGF-β1 (10 ng/ml) for 15min to 24 hours. n≥3/group，**P*<0.05, * * *P*<0.01 compared with control group. Bars represented means ±SEM.

Methods：Analysis of ATP Concentrations

The concentration of intracellular ATP level was measured using ATP Assay Kit (Beyotime Institute of Biotechnology, Nanjing, China) according to the manufacturer's directions. Briefly, After treated with TGF-β1 for 0min, 15min, 30min, 60min, 90min, 2h, 4h, 8h, 12h and 24h, harvested cells were lysed, followed by centrifugation at 4℃, 12,000×g for 5 min. Protein concentration in each group was determined using the BCA protein assay kit. Finally, ATP was determined by mixing 20 μl of the supernatant with 100 μl of luciferase reagent, which catalyzed the light production from ATP and luciferin. Luminescence was measured on a Multiscan Spectrum and quantitated to ATP standards. Standard curve was also generated and total ATP levels were expressed as μmol/μg protein.
